# Assessment of Genotoxicity of Zinc Oxide Nanoparticles Using Mosquito as Test Model

**DOI:** 10.3390/toxics11110887

**Published:** 2023-10-29

**Authors:** Kanwaljit Kaur Ahluwalia, Kritika Thakur, Amrik Singh Ahluwalia, Abeer Hashem, Graciela Dolores Avila-Quezada, Elsayed Fathi Abd_Allah, Neelam Thakur

**Affiliations:** 1 Department of Zoology, Akal College of Basic Sciences, Eternal University, Baru Sahib, Himachal Pradesh 173101, India; kanwaljit58@gmail.com (K.K.A.); kritikapaonta@gmail.com (K.T.); 2 Department of Botany, Akal College of Basic Sciences, Eternal University, Baru Sahib, Himachal Pradesh 173101, India; amrik.s511@gmail.com; 3 Botany and Microbiology Department, College of Science, King Saud University, P.O. Box. 2460, Riyadh 11451, Saudi Arabia; habeer@ksu.edu.sa; 4 Facultad de Ciencias Agrotecnológicas, Universidad Autónoma de Chihuahua, Chihuahua 31350, México; gdavila@uach.mx; 5 Plant Production Department, College of Food and Agricultural Sciences, King Saud University, P.O. Box. 2460, Riyadh 11451, Saudi Arabia; eabdallah@ksu.edu.sa

**Keywords:** chromosomal aberration, *Culex*, genotoxicity, mosquito, nanoparticles

## Abstract

The widespread applications of ZnO NPs in the different areas of science, technology, medicine, agriculture, and commercial products have led to increased chances of their release into the environment. This created a growing public concern about the toxicological and environmental effects of the nanoparticles. The impact of these NPs on the genetic materials of living organisms is documented in some cultured cells and plants, but there are only a few studies regarding this aspect in animals. In view of this, the present work regarding the assessment of the genotoxicity of zinc oxide nanoparticles using the mosquito *Culex quinquefaciatus* has been taken up. Statistically significant chromosomal aberrations over the control are recorded after the exposure of the fourth instar larvae to a dose of less than LD_20_ for 24 h. In order to select this dose, LD_20_ of ZnO NPs for the mosquito is determined by Probit analysis. Lacto-aceto-orcein stained chromosomal preparations are made from gonads of adult treated and control mosquitoes. Both structural aberrations, such as chromosomal breaks, fragments, translocations, and terminal fusions, resulting in the formation of rings and clumped chromosomes, and numerical ones, including hypo- and hyper-aneuploidy at metaphases, bridges, and laggards at the anaphase stage are observed. The percentage frequency of abnormalities in the shape of sperm heads is also found to be statistically significant over the controls. Besides this, zinc oxide nanoparticles are also found to affect the reproductive potential and embryo development as egg rafts obtained from the genetic crosses of ZnO nanoparticle-treated virgin females and normal males are small in size with a far smaller number of eggs per raft. The percentage frequencies of dominant lethal mutations indicated by the frequency of unhatched eggs are also statistically significant (*p* < 0.05) over the control. The induction of abnormalities in all of the three short-term assays studied during the present piece of work indicates the genotoxic potential of ZnO NPs, which cannot be labeled absolutely safe, and this study pinpoints the need to develop strategies for the protection of the environment and living organisms thriving in it.

## 1. Introduction

Nanoparticles (NPs) are particles of matter having a size of less than 100 nanometers (nm). Nanomaterials have already been in use in different industrial, medical, and agriculture sectors for the past decades. Nowadays, the trend of replacing conventional zinc with its nanoparticles is increasing as these NPs have attracted extra attention due to their anti-bacterial, anti-inflammatory, wound healing, and efficient ultraviolet (UV) filtering properties [1,2,3]. That is why their use in consumer products like skin ointments, cosmetics, fabrics, food packaging, insecticides, fertilizers, dental composites, and paints has gradually increased [4,5,6,7,8,9]. These NPs are also utilized in the processing of ceramics and rubber, wastewater treatment, electronic gadgets, and biosensors [10,11,12]. These particles have also been found to play an important role in drug delivery and gene therapy applications in different studies [13,14,15]. The utility of ZnO NPs for drug delivery is attributed to their two important properties, the ability to penetrate through capillaries owing to their nano size and their absorption by the cells, resulting in the accumulation of drugs at the target sites. The levels of ZnO NPs in the environment are increasing constantly due to their extensive applications, and this rise is expected to pose a risk to the non-target aquatic as well as terrestrial organisms, including humans [16,17,18].

Various studies regarding the hazardous effects of ZnO NPs due to their exposure at the production, disposal, and usage level have been reported [19,20,21]. The toxic effects in the form of liver, lung, testis, kidney, and brain damage after exposure of these particles have also been reported [22,23,24,25,26]. The ZnO NPs-induced multiple toxic response was observed in soil organisms, bacteria, algae, plants, nematodes, fish, and mammals [27,28,29,30,31]. It also affects male reproductive system, male fertility, and fetal development [32,33]. 

Due to the ever-growing use of ZnO NPs in most industrial and agricultural products, and in view of different toxicological studies, it becomes essential to investigate the genotoxic potential of these nanoparticles and label these safe/unsafe for biodiversity and human health. Since mosquito species of *Anopheles* and *Culex* have been successfully used as a test material earlier for the screening of heavy metal compounds and complex agrochemicals [34,35,36,37,38,39,40], an attempt has been made to use this organism as a test model for assessing the genotoxicity of ZnO nanoparticles. 

## 2. Materials and Methods

The mosquito, *Culex fatigans* Weidman (Syn *Culex quinquefasciatus* Say), family Culicidae, order Diptera has been used as experimental model. The colony of the species was raised and maintained in rearing cages kept at 75 ± 5% relative humidity and 27 °C ± 2 °C temperature. 

### 2.1. Selection of Dose 

Selection of dose for such studies is important as too large or too small dose can give misleading results. Doses that exhibit toxicity but does not reduce the population of the organism drastically are generally selected for short term assays. That is why LD_20_ (lethal dose 20) of ZnO NPs for the mosquito is determined and selected dose is less than LD_20_ value.

### 2.2. Determination of LD_20_ of ZnO NPs for Mosquito C. quinquefasciatus

**Figure 1:**
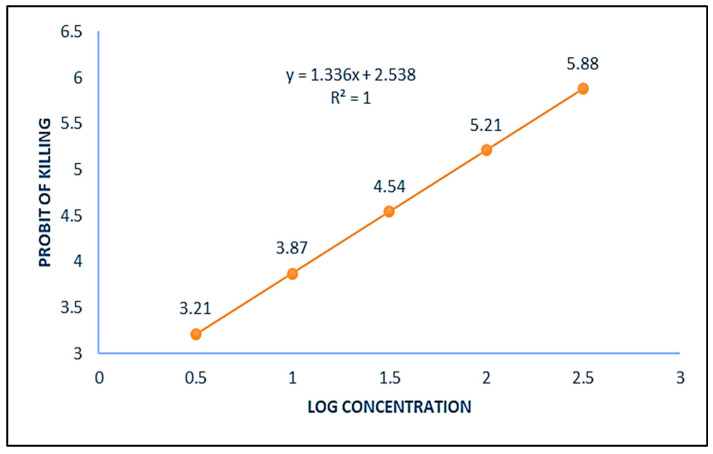
Graph showing relationship between the probit of kill and log of different concentrations of ZnO NPs showing the probit regression line and equation.

Zinc oxide (ZnO) nanoparticles of 50 nm size procured from Department of Genetics, Plant Breeding and Biotechnology, Dr Khem Singh Akal College of Agriculture, Eternal University, Baru Sahib (H.P., India), were used in the present study. A stock suspension of 10 mg/mL was prepared and four more different concentrations of ZnO NPs were made by serial dilutions of the stock. Each dilution reduced the concentration of ZnO NPs ten times. Separate disposable syringes were used for making different concentrations. The suspensions were thoroughly mixed to prevent NPs sedimentation using vortex, just before the treatments. The mosquito toxicity assay was performed by exposing fourth instar larvae to five different concentrations of zinc oxide nanoparticles for 24 h and percent mortality (%) till the formation of adults was recorded (Table 1). From these data, the regression line and equation (Figure 1) were prepared as per Probit analysis [41] and LD_20_ of ZnO NPs for mosquito *C. quinquefasciatus* was calculated, which came out to be 16.46 µg/mL. Among the different concentrations used in mortality test, the dose 10 µg/mL, which is the nearest concentration less than the calculated value of LD_20_ dose was selected for different parameters to be performed in the evaluation of possible genotoxic potential of ZnO NPs in mosquito *C. quinquefaciatus*.

### 2.3. Treatment of Larvae with the Selected Dose

Fourth instar larvae were treated with the selected dose of 10 µg/mL for 24 h. Three replicates of 20 larvae each in 10 mL of the selected dose along with the control comprised of 20 larvae in 10 mL of distilled water were performed. After 24 h of treatment, the larvae were shifted to distilled water, to which a pinch of larval feed (dog biscuits powder and yeast powder in 3:1) was added.

### 2.4. Preparation of Chromosomal Slides

The chromosomal preparations were made from gonads of nearly 12 h old adults developed from treated and control larvae using the technique of Crozier [42] with slight modifications. The colchicine (C_22_H_28_NO_8_) molecular weight 399.44 extra pure quality (98.6%) supplied by Loba Chemie Pvt. Ltd., Mumbai, India was used to arrest the dividing cells at metaphase stage. The adult mosquitoes were fed on cotton pads soaked in 0.1% colchicine and sprinkled with a pinch (1–2 mg) of glucose. After 2 h of colchicine treatment, gonads were dissected out in 1% sodium citrate solution, fixed in Carnoy’s fixative (3 ethanol:1 acetic acid) for 5–7 min, and then were stained in 2% lacto-aceto-orcein stain. 

### 2.5. Sperm Head Assay

For this, sperm smears from the testes of treated and control adult male mosquitoes in 1% sodium citrate solution were made. The smears were stained in 2% lacto-aceto-orcein stain. The slides were studied for sperm head abnormalities.

### 2.6. Dominant Lethal Test

**Figure 2:**
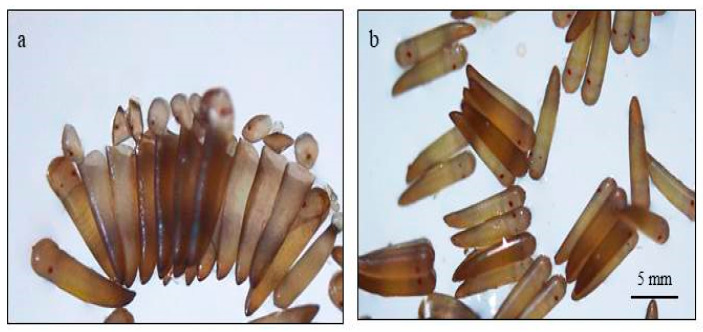
(**a**) Hatched and (**b**) unhatched eggs.

The normal males were crossed with the treated virgin females in a separate rearing cage. The egg rafts obtained were kept in rearing bowls containing distilled water for hatching. After 3 days of hatching, the egg rafts were examined under a stereoscopic binocular. The eggs with open operculum were counted as hatched and those with intact operculum as unhatched (Figure 2). The percent frequency of unhatched eggs was used as the measure of the dominant lethality.

The data collected in all three parameters performed were analyzed statistically by analysis of variance (ANOVA) using SPSS 25.0 (SPSS Inc., Chicago, IL, USA) software. Statistical significance was calculated at level *p* < 0.05. 

## 3. Results

### 3.1. Chromosomal Aberrations

**Figure 3:**
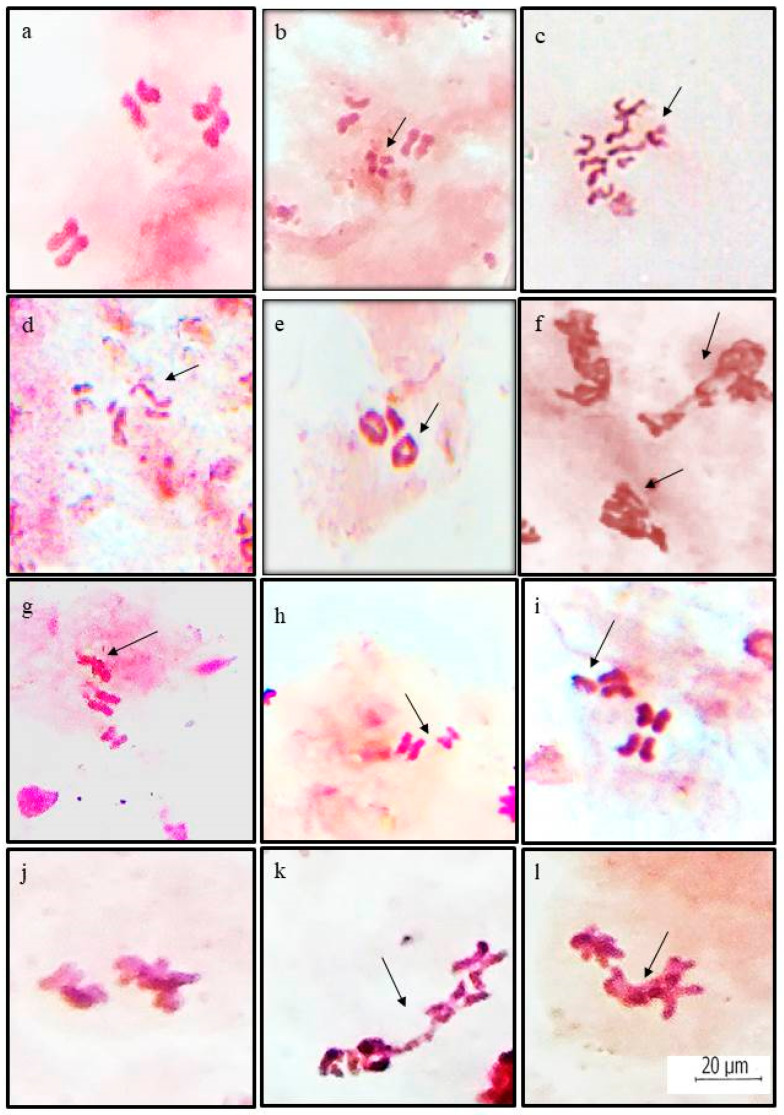
Different types of structural and numerical chromosomal aberrations induced by ZnO nanoparticles in gonadial cells of *Culex quinquefasciatus* (**a**) normal metaphase; (**b**) chromosomal break; (**c**) chromosomal fragments; (**d**) +−translocation; (**e**) terminal fusions forming rings; (**f**) clumped or sticky chromosomes; (**g**) hypoaneuploid (2n − 1); (**h**) hypoaneuploid (2n − 2); (**i**) hyperaneuploid (2n + 1); (**j**) normal anaphase; (**k**) anaphase with bridge; (**l**) anaphase with laggard.

Both structural as well as numerical chromosomal changes were recorded at metaphase stages in the gonadial cells of mosquito *C. quiquefaciatus* after the treatment of ZnO NPs. Structural changes included chromosomal breaks, fragments, translocations, terminal fusions, and clumped chromosomes, whereas numerical alterations observed were aneuploid cells. Aneuploids encountered were hypoaneuploids cells with 2n − 1 and 2n − 2 chromosomes, as well as hyperaneurploids with 2n + 1 and 2n + 2 chromosomes (Figure 3). At least 80 cells from each of the three replicates were studied in treated and control lots, and percent frequency was calculated from the data collected. In total, 27.64 ± 3.33% chromosomal aberrations (Table 2) were reported to be induced by the nanoparticles as against 4.74 ± 0.72% in control. The percent frequency of structural alterations was calculated to be 21.83 ± 3.04%. The occurrence of cells with clumped chromosomes showed a frequency of 8.33 ± 1.10% and was the most abundant structural aberration. Total numerical aberrations reported were 5.81 ± 1.16 %, and these were 0.41 ± 0.41% only in the control population. 

Beside the above-mentioned chromosomal alterations at metaphase stage, some abnormal cells at the anaphase stage showing chromosomal bridges and laggards were also observed (Figure 3) in slides prepared from the gonads of mosquitoes without colchicine treatment. 

### 3.2. Sperm Head Assay

**Figure 4:**
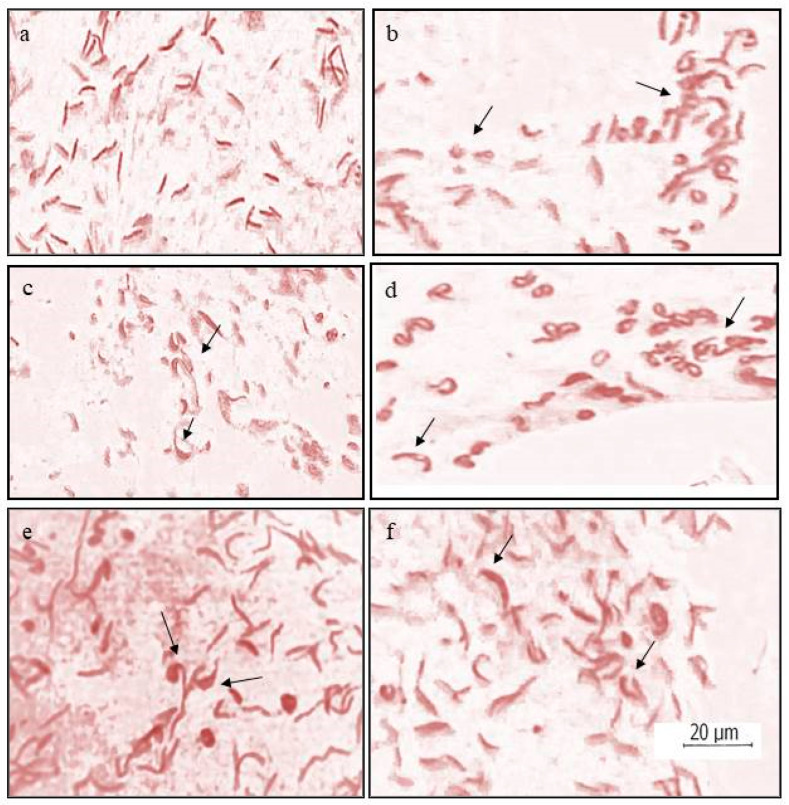
Different types of sperm head abnormalities induced by ZnO NPs in *Culex quinquefasciatus* (**a**) normal sperms; (**b**) twisted; (**c**) sickle shaped and vesicular; (**d**) vacuolated and twisted; (**e**) vacuolated oval and triangular; (**f**) sickle shaped.

In sperm head assay, statistically significant abnormal head shapes were found in treated sperm smears over control. At least 600 sperms were studied in each of the three replicates of treated and control lots. Various sperm head abnormalities, including sickle-shaped, triangular, oval, vesicular, etc. (Figure 4), were observed. The overall percentage of sperm head abnormalities recorded was 11.63 ± 1.99 after treatment with ZnO nanoparticles against 0.93 ± 0.11% in control (Table 3). The analysis of variance showed that the results obtained from the present experiment were statistically significant at *p* < 0.05.

### 3.3. Dominant Lethal Test

For the dominant lethal test, egg rafts obtained from a genetic cross of ZnO nanoparticles treated virgin females and normal males were screened after their hatching for dominant lethal mutations induced, which is indicated by the frequency of unhatched eggs. This is because of the fact that the organism dies during its development within the egg due to dominant lethal mutation, and such eggs with dead embryos do not hatch. At least three egg rafts each were studied from treated and control crosses. The number of unhatched eggs was higher in egg rafts obtained from the treated cross than those of the control cross. Moreover, the fecundity of females, i.e., the potential to lay eggs, decreased as the count of total number of eggs laid per egg raft was observed to be less than the control after treatment (Table 4).

The frequency of dominant lethal mutations induced by the nanoparticles was 12.87 ± 0.679%, which is far more than in the control (Table 5). These results obtained were found to be statistically significant at *p* < 0.05 over control.

## 4. Discussion

Genotoxicity means genetic damage and alterations caused by various chemical and physical agents. Nanomaterials are reported to interact with enzymes involved in the regulation of cell division, thus causing cytotoxic as well as genotoxic effects [43]. This study revealed the genotoxicity of ZnO nanoparticles in *C. quinquefaciatus* at a dose of 10 µg/mL. Our results demonstrated that exposure of 4th instar larvae for 24 h to ZnO NPs induced statistically significant chromosomal aberrations over the controls. Chromosomal breaks, fragments, translocations, and terminal fusions resulting in the formation of rings and clumped chromosomes were the various structural variations observed. The most frequent chromosomal aberration found was the clumping of chromosomes, which could be due to the attainment of a sticky nature after the treatment. The possible reason for the occurrence of these changes may be the removal of the protective telomeres of the chromosomes by zinc oxide nanoparticles, which resulted in their sticky nature, thus causing attachment of a chromosome or its fragment to another one, producing translocations, rings, and clumped mass of chromosomes. Sticky chromosomes were also recorded as the most common aberration at various mitotic stages in onion and garlic root tips treated with ZnO NPs [44]. Similar structural chromosomal alterations were reported in rat bone marrow cells after treatment with ZnO NPs [45]. However, Srivastav and others [46] observed such chromosomal alterations only at a high dose of 2000 mg/kg but not in the rats injected with the low dose of 300 mg/kg. Ickrath et al. [47] indicated the appearance of cytotoxic effects of ZnO NPs at high concentrations of 50 µg/mL and genotoxic effects at even as low concentrations as 1 and 10 µg/mL in human mesenchymal stem cells. Gumus et al. [48] observed chromosome aberrations as well as induction of micronuclei in vitro studies on human peripheral lymphocytes due to the effect of ZnO NPs. This study indicated mutagenic, cytotoxic, and clastogenic effects of these particles in humans after exposure of from 24 to 48 h. Khan and his co-workers [49] also studied the genotoxicity of these particles in human lymphocytes and observed the induction of DNA damage at different concentrations. Motta and others [50] analyzed the muta/genotoxic effects of ZnO NPs and their soluble counterpart (ZnCl_2_) in *Lithobates catesbeianus*. They also noticed significant DNA damage in the tadpoles of this amphibian after their exposure to ZnO NPs at different concentrations for 7 days, as indicated by increased induction of chromosomal aberrations and micronuclear bodies as compared to the control group. Numerical aberrations like hypo- and hyper-aneuploids have also been observed in the present experiments. However, no polyploidy was induced by these nanoparticles. The main cause of aneuploidy could be the non-disjunction of chromosomes during the anaphase stage, resulting in unequal distribution of chromosomes to the daughter cells. This possibility of induction of aneuploids by the nanoparticles was endorsed by our own observation of the occurrence of abnormal anaphases having chromosome bridges and laggards in the treated cells. Similar results regarding the induction of bridges and laggards at anaphase were also reported earlier in root cells of plants exposed to zinc oxide nanoparticles [44,51]. These particles also had a clastogenic and aneugenic impact, which was clearly depicted by chromosomal cytological investigations in root cells of *Vicia faba* by the treatment of ZnO NPs [52]. The presence of these structural and numerical aberrations reflects the genotoxic effect of ZnO NPs. Similar results were also reported by Manzo et al. [18] in *V. faba* root tip cells using the micronucleus test. These nanoparticles could cause clastogenic effects by interfering with the development of mitosis, and it might be due to the inhibition of the S-phase or G_2_ phase of the cell cycle [53]. 

Some in vivo, as well as in vitro studies, have provided significant proof of the toxicity of NPs in male gonadial cells [32,54]. Some other studies have shown the accumulation of the NPs in the testes as well as their ability to cross the blood–testis barriers in mice, and they reported the induction of abnormalities in spermatogonial cells during their maturation by ZnO NPs and suggested this to be the reason of observed abnormal changes in the sperm morphology [55,56]. Such abnormalities in the shapes of sperm heads were also observed during the sperm head assay performed in the present study. Talebi et al. [22] used adult male mice to study the effect of ZnO NPs on epididymal sperms for their morphological changes through testicular histology and reported these particles as testicular toxicants. Earlier, Srivastav and others [46] analyzed the semen of Swiss mice after oral exposure for about a month to two different concentrations of zinc oxide nanoparticles and observed decreased sperm count compared to controls in the high-dose group. Besides this, sperms with abnormal morphology and reduced motility were also reported. 

In the present study, zinc oxide nanoparticles were also found to affect reproductive potential and embryo development as egg rafts obtained from the genetic cross of ZnO nanoparticles, normal males and treated virgin females were small in size with a far smaller number of eggs per egg raft. These egg rafts, when analyzed after their hatching, the percentage frequency of unhatched eggs was statistically significant (*p* < 0.05) over the controls. This is because of the fact that the organism dies during its development within the egg due to dominant lethal mutations induced [36]. These results are supported by the findings of Yadav [57] regarding the retention and deposition of these nanoparticles in different vital organs and causing several abnormalities in reproductive structures, including eggs in nematode *Caenohabditis elegans* after only a twelve-hour exposure to these particles. Embryo toxicity has also been reported in zebrafish exposed to these particles [32].

The induction of chromosomal and sperm head abnormalities in the present piece of work indicates the possibility of the internalization of the nanoparticles into the nuclei of cells by crossing across their plasma and nuclear membranes. This view is supported by the report of Magdolenova and associates [43] who found their transport across the plasma and nuclear membranes by diffusion or through the nuclear pore complexes to affect chromosomal DNA directly. NPs are reported to have been internalized by increasing the membrane permeability of bacterial cells [58]. Ickrath et al. [47] observed intracellular NP accumulation up to 6 weeks. Singh [59] suggested that, after entering into the cells, ZnO nanoparticles aggregates in the Zn^2+^ ion form, and may induce DNA damage either by producing reactive oxygen species (ROS) in cells or associating directly with DNA strand, causing chromosome distortions. DNA damage and oxidative stress have also been reported in the liver cells of mice after it was injected with different concentrations of ZnO NPs of <100 nm size [24,60]. The results obtained by Chang et al. [61] also found these particles of sizes less than 50 nm at a dose of more than 25 μg/mL as cytotoxic and the possible mechanism for this cytotoxicity was suggested to be due to DNA damage, interference with checkpoint kinases resulting in cell cycle arrest, and the production of ROS. The generation of ROS, which in turn causes lipid peroxidation, was responsible for the enhanced intrinsic toxicity of ZnO NPs [62]. The findings of Sharma et al. [63] demonstrated the genotoxic potential of these particles in human epidermal cells even at low concentrations, which was suggested to be mediated through lipid peroxidation and oxidative stress. A significant correlation between the genotoxic effect and oxidative stress, which is measured by the depletion of glutathione, the inhibition of superoxide dismutase, and the generation of reactive oxygen species had been reported by earlier researchers [45,64,65,66,67].

Earlier, Brunner et al. [68], while studying the toxicity of nanoparticles to the cell line cultures of a rodent and humans, suggested that the metal ions released from nanoparticles within the cells as the main causative agent for the genotoxicity of these nanoparticles. In a study performed by Vimercati et al. [69] on two marine crustacean species, the toxic effect was mainly attributed to the Zn ions, conforming to their crucial role in the toxicity of these particles. However, results obtained by Kumari et al. [44] pointed out that the genotoxic effect of ZnO NPs could not be due to released zinc ions alone as they found ZnO NPs more toxic in comparison to Zn^++^ ions, hinting at probable intrinsic nano size related effect. Kononenko and others [70] explained, on the basis of their results that the sole reason for ZnO NPs genotoxicity is not the Zn ions released from the particles in cell culture medium but rather particle size, which determines the physical properties of their dissolution and cellular internalization may also play an important role in it. ZnO NPs induced higher toxicity than ZnO bulk and zinc ions in bacteria [71]. Similar results were also reported in *Drosophila* [72]. Therefore, the genotoxicity of ZnO NPs observed in *C. quinquefaciatus* by causing chromosomal abnormalities, anaphase distortions, and sperm head abnormalities could be due to the nano size of the zinc oxide particles rather than its ions. Further molecular studies can be performed for detecting point mutations at the nucleotide level in addition to confirming the conflict regarding the involvement of zinc ions or the nano size of these particles in the induction of their genotoxicity. 

## 5. Conclusions

The present study regarding the use of mosquito *C. quinquefaciatus* as a test model for the assessment of genotoxicity of zinc oxide nanoparticles using chromosomal aberrations, the sperm head assay, and the dominant lethal test is a novel study. Since ZnO NPs induced statistically significant abnormalities over the controls, even at a dose less than LD_20_ in all the three parameters performed during the study. It can be concluded that these nanoparticles do have a genotoxic potential and cannot be labeled as absolutely safe for their use in various products being consumed/used directly or indirectly by living organisms, including humans. Such studies are important and must be taken into consideration for developing strategies to protect human and environmental health.

## Figures and Tables

**Table 1:** The percent mortality induced by ZnO NPs in mosquito *C. quinquefasciatus*.

S. No.	Dose/Concentration (µg/mL)	No of Larvae Treated (*n*)	No of Insect Killed (r)	Percent Mortality (r/*n* × 100)
1	10,000	40	34	85
2	1000	40	30	75
3	100	40	31	77.5
4	10	40	13	32.5
5	1	40	12	30
6	Control	40	2	5

**Table 2:** Percentage frequency of the metaphase chromosomal aberrations induced by ZnO NPs in gonadial cells of *C. quinquefaciatus*.

Treatment	Chromosomal Aberrations *											
	Structural Aberrations (S)					Numerical Aberrations (N)				Total Structural Aberrations	Total Numerical Aberrations	Total Aberrations
						Aneuploids						
	Breaks	Fragments	Translocations	Clumped Chromosomes	Terminal fusions	(2n − 1)	(2n − 2)	(2n + 1)	(2n + 2)	S	N	S + N
ZnO NPs	3.68 ± 0.78	3.33 ± 0.83	4.08 ± 1.16	8.33 ± 1.10	2.41 ± 0.72	1.16 ± 0.65	1.58 ± 0.33	1.16 ± 1.16	1.91 ± 0.33	21.83 ± 3.04	5.81 ± 1.16	27.64 ± 3.33
Control	0.76 ± 0.76	0.83 ± 0.41	0.83 ± 0.41	1.91 ± 0.33	0	0	0	0.41 ± 0.41	0	4.33 ± 0.41	0.41 ± 0.41	4.74 ± 0.72

* The data are mean ± S.E of three replicates and is statistically significant at *p* < 0.05.

**Table 3:** The percentage frequency of sperm head abnormalities induced by ZnO NPs in *C. quinquefaciatus*.

Treatment	Sperm Head Abnormalities *				
	Sickle Shaped	Oval	Triangular	Vacuolated	Total
ZnO NPs	10.20 ± 1.95	0.99 ± 0.19	0.22 ± 0.05	0.22 ± 0.05	11.63 ± 1.99
Control	0.88 ± 0.14	0.00	0.05 ± 0.05	0.00	0.93 ± 0.11

* The data are mean ± S.E. of three replicates and is statistically significant at *p* < 0.05.

**Table 4:** Analysis of eggs obtained from genetic cross of ZnO NPs-treated virgin females with normal males for dominant lethal mutations (unhatched eggs).

Treatment	Replicates	Total Eggs/Egg Raft	Unhatched Eggs	% of Unhatched Eggs
Control	1	315	7	2.22
	2	287	3	1.04
	3	301	4	1.32
ZnO NPs	1	178	22	12.35
	2	141	17	12.05
	3	239	34	14.22

**Table 5:** Percentage frequency of dominant lethal mutations induced by ZnO NPs preventing the hatching of eggs in *C. quinquefaciatus*.

Treatment	No. of Egg Rafts Counted	% Age Frequency of Unhatched Eggs (Mean ± S.E.)
Control	3	1.53 ± 0.366
ZnO NPs	3	12.87 ± 0.679 *

* The results are statistically significant (*p* < 0.05).

## Data Availability

Not applicable.

## References

[1] Wahab R., Mishra A., Yun S.I., Kim Y.S., Shin H.S. (2010). Antibacterial activity of ZnO nanoparticles prepared via non-hydrolytic solution route. Appl. Microbiol. Biotechnol..

[2] Wang L., Hu C., Shao L. (2017). The antimicrobial activity of nanoparticles: Present situation and prospects for the future. Int. J. Nanomedicine.

[3] Xie J.J., Li H., Zhang T., Song B., Wang X., Gu Z. (2023). Recent Advances in ZnO Nanomaterial-Mediated Biological Applications and Action Mechanisms. Nanomaterials.

[4] Newman M.D., Stotland M., Ellis J.I. (2009). The safety of nanosized particles in titanium dioxide and zinc oxide based sunscreens. J. Am. Acad. Dermatol..

[5] Aydin Sevinç B., Hanley L. (2010). Antibacterial activity of dental composites containing zinc oxide nanoparticles. J. Biomed. Mater. Res. Part B Appl. Biomater..

[6] Espitia P.J.P., Soares N.D.F.F., Dos Reis Coimbra J.S., De Andrade N.J., Cruz R.S., Medeiros E.A.A. (2012). Zinc oxide nanoparticles: Synthesis, antimicrobial activity and food packaging applications. Food Bioprocess Technol..

[7] Mirzaei H., Darroudi M. (2017). Zinc oxide nanoparticles: Biological synthesis and biomedical applications. Ceram. Int..

[8] Rehmanullah M.Z., Inayat N., Majeed A., Rakshit A., Singh H.B., Singh A.K., Singh U.S., Fraceto L. (2020). Application of nanoparticles in agriculture as fertilizers and pesticides: Challenges and opportunities. New Frontiers in Stress Management for Durable Agriculture.

[9] Jiang J., Pi J., Cai J. (2018). The advancing of zinc oxide nanoparticles for biomedical applications. Bioinorg. Chem. Appl..

[10] Raha S., Ahmaruzzaman M. (2022). ZnO nanostructured materials and their potential applications: Progress, challenges and perspectives. Nanoscale Adv..

[11] Singh R., Kumar R., Kumar A., Kumar D., Kumar M. (2021). Low power and stable resistive switching in graphene oxide-based RRAM embedded with ZnO nanoparticles for nonvolatile memory applications. J. Mater. Sci. Mater. Electron..

[12] Alberti S., Basciu I., Vocciante M., Ferretti M. (2021). Experimental and physico-chemical comparison of ZnO nanoparticles’ activity for photocatalytic applications in wastewater treatment. Catalysts.

[13] Vaishnav J., Subha V., Kirubanandan S., Arulmozhi M., Renganathan S. (2017). Green synthesis of zinc oxide nanoparticles by *Celosia argentea* and its characterization. J. Optoelectron. Biomed. Mater..

[14] Dang Y., Guan J. (2020). Nanoparticle-based drug delivery systems for cancer therapy. Smart Mater. Med..

[15] Singh T.A., Das J., Sil P.C. (2020). Zinc oxide nanoparticles: A comprehensive review on its synthesis, anticancer and drug delivery applications as well as health risks. Adv. Colloid Interface Sci..

[16] Nowack B., Bucheli T.D. (2007). Occurrence, behavior and effects of nanoparticles in the environment. Environ. Pollut..

[17] Hu C.W., Li M., Cui Y.B., Li D.S., Chen J., Yang L.Y. (2010). Toxicological effects of TiO_2_ and ZnO nanoparticles in soil on earthworm *Eisenia fetida*. Soil Biol. Biochem..

[18] Manzo S., Rocco A., Carotenuto R., Picione F.D.L., Miglietta M.L., Rametta G., Francia D.I. (2011). Investigation of ZnO nanoparticles’ ecotoxicological effects towards different soil organisms. Environ.Sci. Pollut. Res..

[19] Nohynek G.J., Antignac E., Re T., Toutain H. (2010). Safety assessment of personal care products/cosmetics and their ingredients. Toxicol. Appl. Pharmacol..

[20] Osmond M.J., McCall M.J. (2010). Zinc oxide nanoparticles in modern sunscreens: An analysis of potential exposure and hazard. Nanotoxicology.

[21] Iavicoli I., Leso V., Beezhold D.H., Shvedova A.A. (2017). Nanotechnology in agriculture: Opportunities, toxicological implications, and occupational risks. Toxicol. Appl. Pharmacol..

[22] Talebi A.R., Khorsandi L., Moridian M. (2013). The effect of zinc oxide nanoparticles on mouse spermatogenesis. J. Assist. Reprod. Genet..

[23] Noori A., Karimi F., Fatahian S., Yazdani F. (2014). Effects of zinc oxide nanoparticles on renal function in mice. Int. J. Biosci..

[24] Tang H.Q., Xu M., Rong Q., Jin R.W., Liu Q.J., Li Y. (2016). The effect of ZnO nanoparticles on liver function in rats. Int. J. Nanomedicine.

[25] Pati R., Das I., Mehta R.K., Sahu R., Sonawane A. (2016). Zinc-oxide nanoparticles exhibit genotoxic, clastogenic, cytotoxic and actin depolymerization effects by inducing oxidative stress responses in macrophages and adult mice. Toxicol. Sci..

[26] Rahdar A., Hajinezhad M.R., Bilal M., Askari F., Kyzas G.Z. (2020). Behavioral effects of zinc oxide nanoparticles on the brain of rats. Inorg. Chem. Commun..

[27] Adams L.K., Lyon D.Y., Alvarez P.J. (2006). Comparative eco-toxicity of nanoscale TiO_2_, SiO_2_, and ZnO water suspensions. Water Res..

[28] Xiong D., Fang T., Yu L., Sima X., Zhu W. (2011). Effects of nano-scale TiO_2_, ZnO and their bulk counterparts on zebrafish: Acute toxicity, oxidative stress and oxidative damage. Sci. Total Environ..

[29] Rajput V.D., Minkina T.M., Behal A., Sushkova S.N., Mandzhieva S., Singh R., Movsesyan H.S. (2018). Effects of zinc-oxide nanoparticles on soil, plants, animals and soil organisms: A review. Environ. Nanotechnol. Monit. Manag..

[30] Ye N., Wang Z., Wang S., Peijnenburg W.J. (2018). Toxicity of mixtures of zinc oxide and graphene oxide nanoparticles to aquatic organisms of different trophic level: Particles outperform dissolved ions. Nanotoxicology.

[31] Stałanowska K., Szablińska-Piernik J., Okorski A., Lahuta L.B. (2023). Zinc oxide nanoparticles affect early seedlings’ growth and polar metabolite profiles of pea (*Pisum sativum* L.) and wheat (*Triticum aestivum* L.). Int. J. Mol. Sci..

[32] Choi J.S., Kim R.O., Yoon S., Kim W.K. (2016). Developmental toxicity of zinc oxide nanoparticles to zebrafish (*Danio rerio*): A transcriptomic analysis. PLoS ONE.

[33] Pinho A.R., Rebelo S., Pereira M.D.L. (2020). The impact of zinc oxide nanoparticles on male (in) fertility. Materials.

[34] Sharma G.P., Sobti R.C., Chaudhry A., Gill R.K., Ahluwalia K.K. (1987). Mutagenic potential of a substituted urea herbicide, Monuron. Cytologia.

[35] Sharma G.P., Sobti R.C., Chaudhry A., Ahluwalia K.K. (1988). Genotoxicity of two heavy metal compounds-lead acetate and mercuric chloride in the mosquito, *Anopheles stephensi* Liston (Culicidae: Diptera). Cytologia.

[36] Sharma G.P., Sobti R.C., Chaudhry A., Ahluwalia K.K. (1989). Chromosome aberrations and dominant lethals in *Culex fatigans* due to mercuric chloride. Cytobios.

[37] Sharma G.P., Chaudhry A., Ahluwalia K.K. (1989). Genotoxic effect of three N-oxidized derivatives of o-toluidine on the germ cells of a mosquito, *Culex fatigans* (Culicidae: Diptera). Res. Bull. Panjab Univ. Sci..

[38] Ahluwalia K.K., Chaudhry S. (1999). Genotoxic potential of mercuric nitrate in *Culex quinquefasciatus*. Res. Bull. Panjab Univ..

[39] Ahluwalia K.K., Ahluwalia A.S., Gaur R. (2014). Mutagenic potential of nickel nitrate in mosquito *Culex fatigans*. Perspectives and Trends, Science Technology and Environment.

[40] Marwaha L. (2016). In vivo genotoxicity evaluation of carbaryl pesticides using polytene chromosomes of *Anopheles culicifacies* (Diptera: Culicidae). Toxicol. Int..

[41] Finney D.J., Finney D.J. (1971). A statistical treatment of the sigmoid response curve. Probit Analysis.

[42] Crozier R. (1968). An acetic acid dissociation, air-drying technique for insect chromosomes, with aceto-lactic orcein staining. Stain Technol..

[43] Magdolenova Z., Collins A., Kumar A., Dhawan A., Stone V., Dusinska M. (2014). Mechanisms of genotoxicity. A review of *in vitro* and in vivo studies with engineered nanoparticles. Nanotoxicology.

[44] Kumari M., Khan S.S., Pakrashi S., Mukherjee A., Chandrasekaran N. (2011). Cytogenetic and genotoxic effects of zinc oxide nanoparticles on root cells of *Allium cepa*. J. Hazard. Mater..

[45] Ghosh M., Sinha S., Jothiramajayam M., Jana A., Nag A., Mukherjee A. (2016). Cyto-genotoxicity and oxidative stress induced by zinc oxide nanoparticle in human lymphocyte cells *in vitro* and Swiss albino male mice in vivo. Food Chem.Toxicol..

[46] Srivastav A.K., Kumar A., Prakash J., Singh D., Jagdale P., Shankar J., Kumar M. (2017). Genotoxicity evaluation of zinc oxide nanoparticles in Swiss mice after oral administration using chromosomal aberration, micronuclei, semen analysis, and RAPD profile. Toxicol. Ind. Health.

[47] Ickrath P., Wagner M., Scherzad A., Gehrke T., Burghartz M., Hagen R., Hackenberg S. (2017). Time-dependent toxic and genotoxic effects of zinc oxide nanoparticles after long-term and repetitive exposure to human mesenchymal stem cells. Int. J. Environ. Res. Public Health.

[48] Gümüş D., Berber A.A., Ada K., Aksoy H. (2014). In vitro genotoxic effects of ZnO nanomaterials in human peripheral lymphocytes. Cytotechnology.

[49] Khan M., Naqvi A.H., Ahmad M. (2015). Comparative study of the cytotoxic and genotoxic potentials of zinc oxide and titanium dioxide nanoparticles. Toxicol. Rep..

[50] Motta A.G.C., do Amaral D.F., Benvindo-Souza M., Rocha T.L., Silva D.D.M. (2020). Genotoxic and mutagenic effects of zinc oxide nanoparticles and zinc chloride on tadpoles of *Lithobates catesbeianus* (Anura: Ranidae). Environ. Nanotechnol. Monit. Manag..

[51] Shaymurat T., Gu J., Xu C., Yang Z., Zhao Q., Liu Y. (2012). Phytotoxic and genotoxic effects of ZnO nanoparticles on garlic (*Allium sativum* L.): A morphological study. Nanotoxicology.

[52] Youssef M.S., Elamawi R.M. (2020). Evaluation of phytotoxicity, cytotoxicity, and genotoxicity of ZnO nanoparticles in *Vicia faba*. Environ. Sci. Pollut. Res..

[53] Borboa L., De la Torre C. (1996). The genotoxicity of Zn (II) and Cd (II) in *Allium cepa* root meristematic cells. New Phytol..

[54] Braydich-Stolle L.K., Lucas B., Schrand A., Murdock R.C., Lee T., Schlager J.J., Hofmann M.C. (2010). Silver nanoparticles disrupt GDNF/Fyn kinase signaling in spermatogonial stem cells. Toxicol. Sci..

[55] Borm P.J., Kreyling W. (2004). Toxicological hazards of inhaled nanoparticles–potential implications for drug delivery. J. Nanosci. Nanotechnol..

[56] Tang Y., Chen B., Hong W., Chen L., Yao L., Zhao Y., Xu H. (2019). ZnO nanoparticles induced male reproductive toxicity based on the effects on the endoplasmic reticulum stress signaling pathway. Int. J. Nanomedicine.

[57] Yadav S. (2014). Retention of particles in *Caenohabditis elegans* after exposure to zinc oxide nanoparticles. Int. J. Curr. Microbiol. App. Sci..

[58] Brayner R., Ferrari-Iliou R., Brivois N., Djediat S., Benedetti M.F., Fievet F. (2006). Toxicological impact studies based on *Escherichia coli* bacteria in ultrafine ZnO nanoparticles colloidal medium. Nano Lett..

[59] Singh S. (2019). Zinc oxide nanoparticles impacts: Cytotoxicity, genotoxicity, developmental toxicity, and neurotoxicity. Toxicol. Mech. Methods.

[60] Sharma V., Singh P., Pandey A.K., Dhawan A. (2012). Induction of oxidative stress, DNA damage and apoptosis in mouse liver after sub-acute oral exposure to zinc oxide nanoparticles. Mutat. Res. Genet. Toxicol. Environ. Mutagen..

[61] Chang M., Tang C., Lin Y., Liu H., Wang T., Lan W., Cheng R., Lin Y., Chang H., Jeng J. (2021). Toxic mechanisms of Roth801, canals, microparticles and nanoparticles of ZnO on MG-63 osteoblasts. Mater. Sci. Eng. C..

[62] Jeng H.A., Swanson J. (2006). Toxicity of metal oxide nanoparticles in mammalian cells. J. Environ. Sci. Health A..

[63] Sharma V., Shukla R.K., Saxena N., Parmar D., Das M., Dhawan A. (2009). DNA damaging potential of zinc oxide nanoparticles in human epidermal cells. Toxicol. Lett..

[64] Schiavo S., Oliviero M., Miglietta M., Rametta G., Manzo S. (2016). Genotoxic and cytotoxic effects of ZnO nanoparticles for *Dunaliella tertiolecta* and comparison with SiO_2_ and TiO_2_ effects at population growth inhibition levels. Sci. Total Environ..

[65] Ng C.T., Yong L.Q., Hande M.P., Ong C.N., Yu L.E., Bay B.H., Baeg G.H. (2017). Zinc oxide nanoparticles exhibit cytotoxicity and genotoxicity through oxidative stress responses in human lung fibroblasts and *Drosophila melanogaster*. Int. J. Nanomed..

[66] Singh R., Cheng S., Singh S. (2020). Oxidative stress-mediated genotoxic effect of zinc oxide nanoparticles on *Deinococcus radiodurans*. 3 Biotech..

[67] Heim J., Felder E., Tahir M.N., Kaltbeitzel A., Heinrich U.R., Brochhausen C., Brieger J. (2015). Genotoxic effects of zinc oxide nanoparticles. Nanoscale.

[68] Brunner T.J., Wick P., Manser P., Spohn P., Grass R.N., Limbach L.K., Stark W.J. (2006). In vitro cytotoxicity of oxide nanoparticles: Comparison to asbestos, silica, and the effect of particle solubility. Environ. Sci. Technol..

[69] Vimercati L., Cavone D., Caputi A., De Maria L., Tria M., Prato E., Ferri G.M. (2020). Nanoparticles: An experimental study of zinc nanoparticles toxicity on marine crustaceans. General overview on the health implications in humans. Front. Public Health.

[70] Kononenko V., Repar N., Marušič N., Drašler B., Romih T., Hočevar S., Drobne D. (2017). Comparative in vitro genotoxicity study of ZnO nanoparticles, ZnO macroparticles and ZnCl_2_ to MDCK kidney cells: Size matters. Vitr. Toxicol..

[71] Jiang W., Mashayekhi H., Xing B. (2009). Bacterial toxicity comparison between nano—and micro-scaled oxide particles. Environ. Pollut..

[72] Carmona E.R., Inostroza B.C., Rubio L., Marcos R. (2016). Genotoxic and oxidative stress potential of nanosized and bulk zinc oxide particles in *Drosophila melanogaster*. Toxicol. Ind. Health.

